# *Gochujang* suppresses cell survival and changes reactive oxygen species metabolism in colorectal cancer cells

**DOI:** 10.29219/fnr.v68.10844

**Published:** 2024-10-21

**Authors:** Eun-Bi Seo, So-Min Oh, Anna Han

**Affiliations:** 1 Department of Food Science and Human Nutrition, Jeonbuk National University, Jeonju, Republic of Korea; 2 K-food Research Center, Jeonbuk National University, Jeonju, Republic of Korea

**Keywords:** Gochujang, colorectal cancer, anticancer, mechanism, reactive oxygen species

## Abstract

There is a significant global increase in colorectal cancer (CRC) among young adults. *Gochujang*, one of the signature Korean traditional fermented foods, contains various bioactive compounds and has multiple health-beneficial effects, including anticancer effects; however, the detailed cellular and molecular mechanisms of its anticancer outcomes are not fully understood. The objective of the present study was to investigate the detailed underlying anticancer mechanisms of *Gochujang* in CRC cells. *Gochujang* was extracted with 80% ethanol, and total polyphenol contents (9.9 ± 1.63 mgGAE/g) and total flavonoid contents (0.14 ± 0.07 mgQE/g) of *Gochujang* extract (GE) were evaluated. GE significantly suppressed cell viability, migration, and colony formation in CRC cells. Also, GE increased the cell cycle arrest-related protein p21 level, whereas it decreased cell cycle progression-associated proteins, such as p-Rb. Moreover, GE markedly elevated the levels of proapoptotic proteins (e.g. Bim and c-PARP), while it downregulated antiapoptotic protein expressions (e.g. Bcl-2 and Bcl-xL). GE also altered the expression of the autophagy-involved proteins. Furthermore, GE strongly reduced the expression of major antioxidant enzymes and increased the reactive oxygen species (ROS) generation in CRC cells, causing an imbalance of ROS metabolism. In conclusion, this study demonstrated that *Gochujang* exerts anticancer effects in CRC cells by inhibiting cell proliferation, increasing cell death, and interrupting ROS metabolism.

## Popular scientific summary

- *Gochujang* extract (GE) shows an anticancer effect by decreasing cell viability, migration ability, and colony formation in colorectal cancer (CRC) cells.
- GE downregulates the protein expressions associated with cell proliferation and upregulates the protein expressions related to cell cycle arrest in CRC cells.
- GE induces cell death by regulating the protein levels related to apoptosis and autophagy.
- GE causes the imbalance of ROS metabolism via the reduction of antioxidant enzyme expression and elevation of ROS production.

Colorectal cancer (CRC) is the third most common cancer in South Korea, and the incidence of CRC has been steadily increasing in recent years, especially among young adults (1–3). Especially among young adults, because of their dietary patterns, there has been a dramatic increase in gastrointestinal (GI) tract health issues, including inflammatory bowel disease (IBD) and constipation, elevating the CRC risk, subsequently (4, 5). Indeed, according to the CRC screening guideline by the National Cancer Center in Korea, colon microscopy is now recommended for adults ≥45 years to screen for polyps and CRC risk (6). There are multiple causal factors of CRC, including diet (7). For example, excessive energy intake from the Western diet (e.g. high-fat diet) leads to obesity and/or metabolic disorders, contributing to CRC development (8). Furthermore, the consumption of frequent red and processed meat is also associated with an increased risk of CRC (9, 10). Several aromatic compounds found in red and processed meat alter DNA or stimulate the bile acids secretion, leading to mutations of colonocytes and CRC development, subsequently (11). In contrast, a plant-rich diet is associated with a lower CRC risk (12). Indeed, diverse bioactive compounds in plant foods exert cancer-preventive and/or ameliorative effects (13). Therefore, maintaining a healthy diet is critical to prevent CRC development and ameliorate CRC.

Research regarding the anticancer effects of bioactive compounds and certain foods has been mainly focused on the inhibition of cancer cell proliferation and the induction of cell death (14, 15). Most of the plant foods-derived bioactive compounds having anticancer effects not only directly induce apoptosis and/or autophagy of cancer cells but also inhibit cell proliferation and/or cell cycle progression (16, 17). Additionally, bioactive compounds and/or foods impact cancer cell metabolism, such as reactive oxygen species (ROS) metabolism (18). ROS, the free radicals containing oxygen, are primarily generated in the mitochondria. Appropriate ROS levels act as essential for cell growth, but excessive levels of ROS cause oxidative stress within the cells, causing the development of various diseases, including cancer (19). Since ROS can be produced during the normal metabolic status, the cells regulate the antioxidant system to remove excessive ROS and maintain appropriate ROS levels, avoiding oxidative stress induction (20). Interestingly, most cancer cells have altered ROS metabolism compared with normal cells, supporting their aggressive growth (20). Indeed, cancer cells have deficient mitochondrial oxidative metabolism, leading to their higher levels of ROS compared to normal cells (20–22). Therefore, targeting the ROS metabolism of cancer cells can be a potential therapeutic approach, as disruption of cancer cell-specific ROS metabolism leads to cancer cell death (21, 23, 24).

Korean traditional fermented foods (KTFFs) contain multiple health-beneficial microorganisms and bioactive compounds, eliciting diverse advantages for health (25–30). Many studies have demonstrated that KTFFs, such as *Gochujang*, *Doenjang*, *Ganjang*, and *Cheonggukjang*, have anticancer effects, anti-obesity effects, anti-inflammatory effects, and antioxidant effects (25, 27, 31–34). For example, during fermentation, *Gochujang* generates multiple probiotics (e.g. *Escherichia coli* and *Lactobacillus reuteri*), allowing benefits in gut microbiota composition and supporting gut health (35). Furthermore, capsaicin, one of the main components of *Gochujang*, plays a major role in its anti-obesity effects (36). Moreover, *Gochujang* ameliorates hepatic inflammation and lipid metabolism and also reduces body weight gain in high-fat diet-induced obese mice (25). Additionally, the anticancer effects of *Gochujang* extracted with 95% methanol were evaluated on gastric, colorectal, and lung cancer cells using the 3-(4,5-Dimethylthiazol-2-yl)-2,5-diphenyltetrazolium bromide (MTT) assay (37). The research regarding the anticancer effects of *Gochujang* extract (GE), including CRC cells, has simply evaluated via cell viability assay with several cancer cell lines (31); however, it is still unclear the detailed cellular and molecular mechanisms of its outcomes. Moreover, the effects of *Gochujang* on ROS metabolism, including the antioxidant system in CRC cells, have not yet been investigated.

Therefore, the first aim of the current study was to evaluate the anticancer effects of GE, especially with its underlying cellular and molecular mechanisms in CRC cells. Additionally, the second aim of the present study was to investigate the impacts of GE on ROS metabolism in CRC cells.

## Materials and methods

### Preparation of GE

*Gochujang* provided by Ganghwa-gun, Sunchang-gun, and Yeongwol-gun in South Korea was mixed and then extracted. 1 L of 80% ethanol was added to the freeze-dried and ground *Gochujang* mixed sample and stirred at room temperature. The supernatant was collected without centrifugation, and 80% ethanol was added to the remaining pellets in a 10× ratio. The mixture was stirred again to obtain the secondary supernatant. The residue was filtered using a vacuum filter, concentrated using a rotary evaporator, and then subjected to freeze-drying for approximately 4 days to obtain a powder, which was subsequently stored at -70°C. For the treatment of cells, the frozen GE sample was mixed with Dimethyl sulfoxide (DMSO) diluted with 1% in Phosphate buffered saline (PBS). The mixture was filtered through a 0.45 μm filter before being used for cell treatment.

### Cell culture

HCT116 and HT29 cells were cultured in RPMI-1640 MEDIUM (HyClone, U.S.A.), and Caco2 cells were cultured in MEM MEDIUM (Gibco, U.S.A.). The media contained 100 IU/mL of penicillin, 100 μg/mL of streptomycin (SIGMA, U.S.A.), and 10% fetal bovine serum (which was heat-inactivated in the water bath) (Gibco, U.S.A.). All cell lines were maintained in an incubator at 37°C in a humidified atmosphere (95% air, 5% CO_2_).

### Radical scavenging activity of GE

Antioxidant assay was measured by 2,2′-Azino-bis (3-ethylbenzthiazoline-6-sulfonic acid) (ABTS) radical scavenging activity and 2,2-diphenyl-1-picrylhydrazyl (DPPH) radical scavenging activity. The ABTS and DPPH radical scavenging activity assays were performed according to a modification of the method of Ehiobu et al. (38). The ABTS was produced by mixing 7.4 mM ABTS and 2.45 mM potassium persulfate prepared in distilled water. The solution was kept in the dark at room temperature overnight. After that, the solution was diluted to achieve an absorbance of 0.7–0.8 nm, then 180 μL ABTS solution and 20 μL of the GE samples or Trolox standard were mixed per well in a 96-well plate, and the mixture was allowed to react in the dark for 10 min. Absorbance was measured at 734 nm. The 0.2 mM DPPH solution in ethanol was prepared in the dark. The 160 μL DPPH solution and 40 μL of the GE samples or Trolox standard were mixed per well in a 96-well plate, and the mixture was allowed to react in the dark for 35 min. Absorbance was read at 517 nm. The absorbance was measured by using the i-control 2.0 software of the microplate reader (TECAN, INFINITE M PLEX).

### Determination of total polyphenol and total flavonoid contents in GE

Total polyphenol content was based on the Singleton et al. (39), with some modifications, and the total flavonoid content was determined by referring to the experimental method of Zhishen et al. (40). To measure the total polyphenol content, a mixture of 10 μL GE sample, 200 μL of sodium carbonate, and 10 μL of 50% 2 N Folin was reacted in the dark at room temperature for 30 min. Absorbance was measured at 760 nm. Gallic acid was used as a standard to determine the total polyphenol content. To determine the total flavonoid content, a mixture of 20 μL GE sample, 80 μL ethanol, and 6 μL of 5% sodium nitrite was reacted at room temperature for 5 min. 12 μL of 10% aluminum chloride hexahydrate was added and reacted at room temperature for 6 min. Finally, 40 μL of 1 N Sodium Hydroxide was added and reacted at room temperature for 11 min. Absorbance was read at 420 nm. Quercetin was used as the standard to measure the total flavonoid content. Absorbance read by i-control 2.0 software of the microplate reader (TECAN, INFINITE M PLEX).

### Cell viability

To assess the toxicity of GE on CRC cells, the EZ-CYTOX kit (DoGenBio, Seoul, Korea) was utilized to measure cell proliferation and cytotoxicity. Cells (HCT116: 1 × 10^3^ cells/well and Caco2: 4 × 10^3^ cells/well) were seeded in a 96-well plate (SPL, US). After allowing the cells to adhere to the plate for 24 h, the media was removed, and the cells were treated with GE in different concentrations (0, 0.25, 0.5, 1, 2, 4, 8, and 10 mg/mL) for 24, 48, and 72 h. After confirmation of IC_50_ of each cell line, GE was treated in different concentrations based on the cell lines and further experimental designs. After the GE treatment, the treated media was removed, and the EZ-CYTOX reagent was mixed with culture media in a 1:10 ratio. Each well was treated with 100 μL of the mixture. The plate was incubated for 1 h and then gently shaken, and the absorbance was measured at 450 nm using the i-control 2.0 software of the microplate reader (TECAN, INFINITE M PLEX). Cell viability was calculated relative to the control (CON).

### Migration assay

The CRC cells (HCT116: 5.5 × 10^4^ cells/mL, Caco2: 6 × 10^4^ cells/mL, and HT29: 8 × 10^4^ cells/mL) were seeded in a 6-well plate (SPL, Pocheon-si, KR). After 24 h, cells were attached to the bottom of the plate, and the center of the bottom of the plate was scratched by sterilized pipette tips. After that, the media was removed, and the cells were treated with GE (0, 0.25, 0.5, and 1 mg/mL) for 72 h. The CON and treatment media were replaced daily. After the treatment, the cells were taken pictures at 0, 24, 48, and 72 h using EVOS™ M7000 Imaging System (ThermoFisher, AMF7000). The images were analyzed using the Image J software to measure the average distance between cells.

### ROS assay

ROS level was measured with the Cellular ROS Assay Kit (ab113851; Abcam, USA) based on the manufacturer’s protocol. HCT116 cells were seeded into 96-well culture plates (SPL) at a density of 1 × 10^4^ cells/well, and Caco2 cells were 2 × 10^4^ cells/well. After the cells adhered for 24 h, GE was treated (0, 0.5, and 2 mg/mL) for 24 h. 4 h before the treatment endpoint, 150 μM tert-butyl hydroperoxide (TBHP), the positive control (P.C.), was added. 45 min before the endpoint, 20 μM 2, 7-dichlorodihydrofluorescein diacetate (DCFDA) was incubated in the dark. Fluorescence was measured with excitation/emission spectra at 485/535 nm using the i-control 2.0 software of the microplate reader (TECAN, INFINITE M PLEX). Fluorescence images were captured using EVOS™ M7000 Imaging System (ThermoFisher, AMF7000).

### Western blot

Western blot analysis was performed with reference to Lee et al. (25). After the GE treatment (for cell death and ROS-related assays, HCT116: 0, 2, and 4 mg/mL, and Caco2: 0, 4, and 8 mg/mL; for migration-related assay, HCT116: 0, 0.25, and 1 mg/mL, and Caco2: 0, 0.5, and 1 mg/mL), the HCT116 and Caco2 cell pellets were lysated and prepared by using 10× RIPA buffer (#9806S, Cell Signaling) diluted by distilled water to 1× lysis buffer with protease inhibitor cocktail (EMD Millipore Corp., Billerica, MA, USA) and phosphatase inhibitor cocktail (EMD Millipore Corp., Burlington, MA, USA). The supernatant was centrifuged at 15,000 g for 20 min. Equal amounts of proteins (HCT116: 30 μg and Caco2: 40 μg) were separated on 8–15% sodium dodecyl-sulfate (SDS) gel. The following primary antibodies were used: β-actin (#A2066) from Sigma-Aldrich Inc. (Gangnam-gu, Seoul); glutathione peroxidase 1/2 (Gpx1/2) (#sc-133160), heme oxygenase 1 (HO-1) (#sc-390991), and matrix metallopeptidase 9 (MMP-9) (#sc-13520) from SANTA CRUZ Biotechnology, Inc. (Dallas, TX, USA); phosphorylated signal transducer and activator of transcription 3 (p-STAT3) (#4113), STAT3 (#30835), p21 (#37543), Rb (#9309), p-Rb (#8516), Cyclin B1 (#4138), cyclin-dependent kinase 2 (CDK2) (#2546), poly (ADP-ribose) polymerases (PARP) (#9542), B-cell lymphoma 2 (Bcl-2) (#3498), B-cell lymphoma-extra large (Bcl-xL) (#2764), Bcl-2 Interacting Mediator of cell death (Bim) (#2933), phosphorylated mechanistic target of rapamycin (p-mTOR) (#2971), mTOR (#2972), microtubule-associated protein 1A/1B-light chain 3 (LC3B) (#2775), nuclear factor erythroid 2-related factor 2 (Nrf2) (#20733), occludin (#91131), kelch-like ECH-associated protein 1 (KEAP1) (#8047), superoxide dismutase 1 (SOD1) (#37385), superoxide dismutase 2 (SOD2) (#13141), and Catalase (#14097) from Cell Signaling Technology, Inc. (Danvers, Massachusetts, USA). Secondary antibody (1:2,000, anti-rabbit or anti-mouse) from Cell Signaling Technology, Inc. (Danvers, Massachusetts, USA) attached 2 h at room temperature and washed with TBS-T. The signal was detected by ECL solution (BIO-RAD, US), and expression was supported by a ChemiDoc KwikQuant imaging system (SCINOMICS, Korea).

### Colony formation assay

CRC cells were seeded with 1,000 cells in 6-well plates and incubated at 37°C overnight. The following day, cells were treated with GE (HCT116 and HT29: 0, 0.25, 0.5, and 1 mg/mL, Caco2: 0, 0.5, 1.5, and 2.5 mg/mL). The treated medium was refreshed every 2 days. After 10 days of cultivation, each well was gently washed twice with PBS, and the colonies were stained with 0.2% crystal violet solution for 2 h at room temperature. Then, the wells were washed three times using distilled water. Finally, the counts of recognized colonies were imaged, and the results were expressed by Image J software.

### Statistical analysis

All data from *in vitro* experiments were performed in triplicate and presented as the mean and standard error of the mean (SEM) of three independent experiments (*n* = 3). Statistical significance was calculated using Student’s *t*-test, and determined at *P < 0.05, **P < 0.01, ***P < 0.001.

## Results

### GE has antioxidant effects

Before the evaluation of GE on CRC cell viability, the antioxidant capacities of GE were measured. Radical scavenging effects in the ABTS and DPPH analysis to confirm radical scavenging ability, the concentration of GE was 0.25–10 mg/mL. The ABTS result was 2.6–86.9%, and the DPPH result was 2.3–17.4% (Supplementary Fig. 1A).

Total phenolic and flavonoid contents were measured as 9.97 mgGAE/g for the total polyphenol contents and 0.14 mgQE/g for the total flavonoid contents based on the GE concentration of 10 mg/mL (Supplementary Fig. 1B).

### GE reduces the CRC cell viability in a time- and dose-dependent manner

**Fig. 1:**
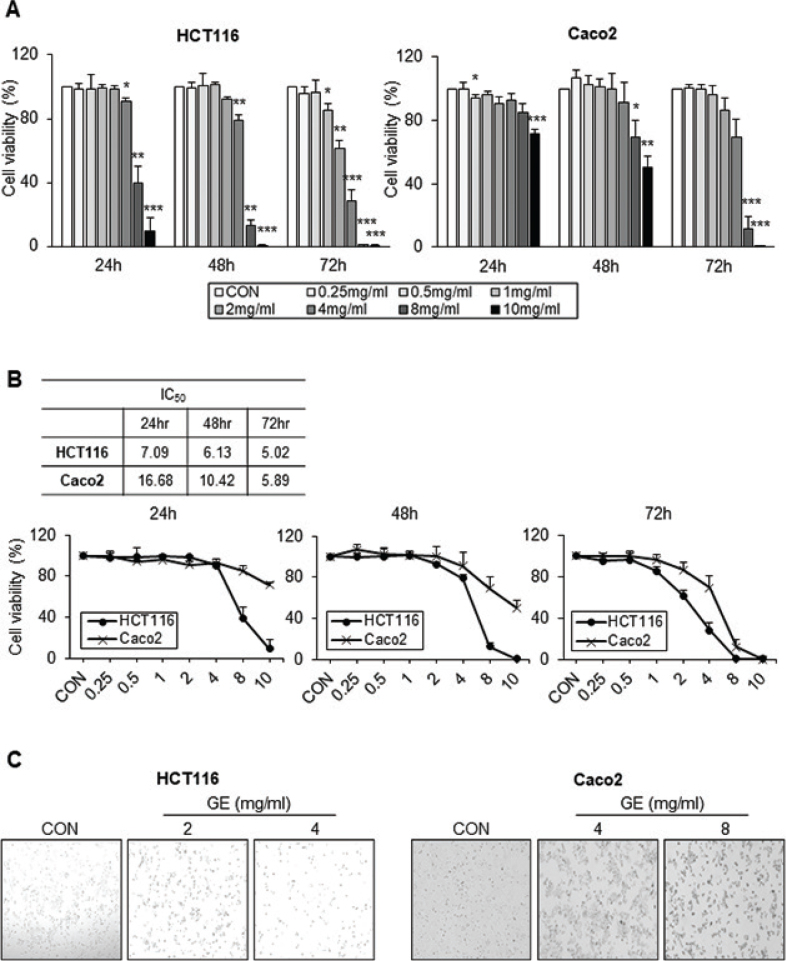
*Gochujang* extract (GE) reduced cell viability and caused cytotoxicity in colorectal cancer (CRC) cells. (A) Cytotoxicity (cell viability) assay in HCT116 and Caco2 cell lines. (B) IC_50_ of CRC cells in GE. After 24 h of cell seeding, cells were treated with GE at 0, 0.25, 0.5, 1, 2, 4, 8, and 10 mg/mL for 24, 48, and 72 h. (C) Morphological images of GE-treated HCT116 cells and Caco2 cells. HCT116 cells were treated with GE at 0, 2, and 4 mg/mL for 48 h. Caco2 cells were treated with GE at 0, 4, and 8 mg/mL for 48 h. Cytotoxicity assay and results were expressed as a % of viable cells compared to CON. Values are mean ± SEM (standard error of the mean) (*n* = 3). *t*-test was performed to evaluate the statistical significance of differences. * (*P* < 0.05), ** (*P* < 0.01), *** (*P* < 0.001).

The effects of GE on CRC cell viability were conducted at different times and concentrations. GE significantly reduces the viability of HCT116, Caco2, and HT29 cells (Fig. 1A and Supplementary Fig. 2A). Additionally, HCT116 cells were the most sensitive to GE among the three CRC cell lines (Fig. 1B and Supplementary Fig. 2A).

The IC_50_ of HCT116 was 7.09, 6.13, and 5.02 mg/mL at 24, 48, and 72 h, respectively; and with Caco2, the IC_50_ was 16.68, 10.42, and 5.89 mg/mL at 24, 48, and 72 h, respectively (Fig. 1B). The IC_50_ in HT29 was 11.82, 8.16, and 6.92 mg/mL at 24, 48, and 72 h (Supplementary Fig. 2A). Based on the different IC_50_ of cell lines, GE treatment concentration was established for the next experiments (Fig. 1C and Supplementary Fig. 2B). In conclusion, GE significantly reduces the cell viability of CRC cell lines time- and dose-dependent manner.

### GE suppresses the migration and colony formation of CRC cells

**Fig. 2:**
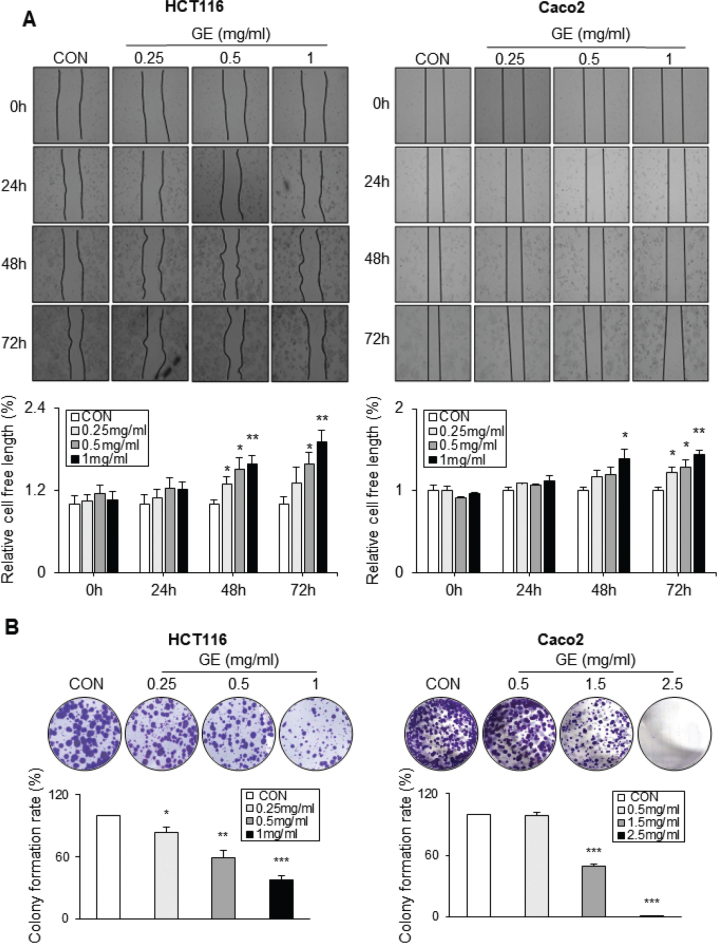
*Gochujang* extract (GE) suppressed the migration and colony formation ability of colorectal cancer (CRC) cells. (A) Scratch/wound-healing assay (migration assay) on CRC cells (HCT116, Caco2). Cells were treated with GE at concentrations of 0, 0.25, 0.5, and 1 mg/mL. Scratch/wound-healing area was measured (0, 24, 48, and 72 h). Migration images of HCT116 and Caco2, and quantification bar graphs of relative cell-free length (%). (B) Six-well plate images and histogram of colony formation rate in response to the GE treatment. HCT116 cells were treated with GE (0, 0.25, 0.5, and 1 mg/mL), and Caco2 cells were treated with GE (0, 0.5, 1.5, and 2.5 mg/mL) for 10 days. Colony formation ability was expressed as % of CON. Values are mean ± SEM (standard error of the mean) (*n* = 3). *t*-test was performed to evaluate the statistical significance of differences. * (*P* < 0.05), ** (*P* < 0.01), *** (*P* < 0.001).

As GE markedly reduces cell viability, the effect of GE on CRC cell migration capacity was evaluated by performing a wound-healing assay. Compared with CON, GE significantly decreases migration of HCT116, Caco2, and HT29 cells time- and dose-dependently (Fig. 2A and Supplementary Fig. 2C). Additionally, GE reduces the expression of MMP-9, a migration-related protein in CRC cells (Supplementary Fig. 3A and 3B).

Next, the effect of GE in colony formation of CRC cell lines was tested. Compared to CON, GE significantly inhibits colony formation dose-dependently in all three CRC cell lines (Fig. 2B and Supplementary Fig. 2D). In summary, GE strongly reduces the migration and colony formation in CRC cells.

### GE changes the protein expression related to cell proliferation, apoptosis, and autophagy in CRC cells

**Fig. 3:**
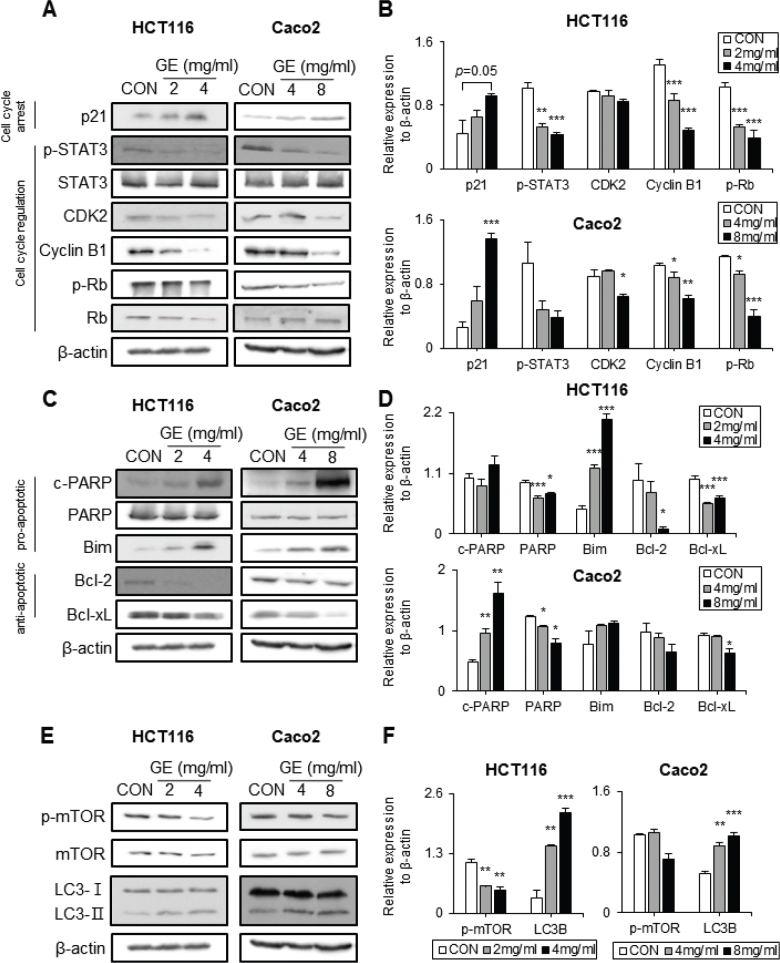
*Gochujang* extract (GE) altered the expression of cell cycle-, apoptosis-, and autophagy-related proteins. (A) Western blot bands of cell cycle arrest or regulatory proteins. (B) Relative protein expression of cell cycle. (C) Western blot bands of proteins associated with apoptosis. (D) Relative protein expression of apoptosis. (E) Western blot bands of proteins associated with autophagy. (F) Relative protein expression of autophagy. HCT116 cells were treated with GE at 0, 2, and 4 mg/mL, and Caco2 cells were treated with GE at 0, 4, and 8 mg/mL for 48 h. Values are mean ± SEM (standard error of the mean) (*n* = 3). *t*-test was performed to evaluate the statistical significance of differences. * (*P* < 0.05), ** (*P* < 0.01), *** (*P* < 0.001). β-Actin was used as a loading control.

Based on the effects of GE on the cell viability, migration, and colony formation results, the underlying cellular and molecular mechanism was investigated by probing the protein expression related to cell cycle arrest, cell cycle regulation, and cell death. p21 is a protein that induces cell cycle arrest, while p-STAT3, CDK2, Cyclin B1, and p-Rb are proteins involved in cell cycle activation (41). As shown in Fig. 3A and 3B, GE significantly upregulated the level of p21, the cell cycle arrest protein in both CRC cell lines. However, GE markedly downregulated the protein levels related to cell proliferation and cell cycle regulation, including p-Rb and cyclin B1, in both CRC cell lines. In addition, GE reduced p-STAT3 in HCT116 with statistical significance, but in Caco2 cells, GE decreased p-STAT3 without statistical significance. The level of CDK2 tended to decrease in HCT116 cells by GE, and Caco2 cells showed a significant reduction of CDK2 level at a high dose of GE (Fig. 3A and 3B).

Next, the effects of GE on cell death-related proteins were probed (Fig. 3C and 3D). In apoptosis, one of the main mechanisms of cell death, Bim and c-PARP proteins are known markers of pro-apoptosis, while Bcl-2 and Bcl-xL are known as antiapoptotic proteins (42). In HCT116 cells, GE significantly increased Bim expression, while cleaved PARP was slightly increased. Moreover, GE significantly decreased the levels of PARP, Bcl-2, and Bcl-xL in HCT116 cells. With Caco2 cells, GE significantly increased the expression of cleaved PARP, and the Bim without statistical significance. Additionally, GE decreased the levels of PARP and Bcl-xL with statistical significance, and Bcl-2 without statistical significance (Fig. 3C and 3D).

GE also significantly altered protein expression related to autophagy. mTOR inhibits cellular autophagy when it is phosphorylated, while LC3B is known as the indicator of autophagy (43). GE significantly decreased p-mTOR levels in HCT116 and resulted in a reduced tendency of p-mTOR in Caco2 (Fig. 3E and 3F). Moreover, GE significantly upregulated the level of LC3B in both cell lines (Fig. 3E and 3F).

In summary, GE upregulates cell cycle arrest-related proteins, downregulates cell proliferation-related proteins, and also elevates apoptosis- and autophagy-related proteins in CRC cells.

### GE disrupts the antioxidant system and ROS metabolism in CRC cells

**Fig. 4:**
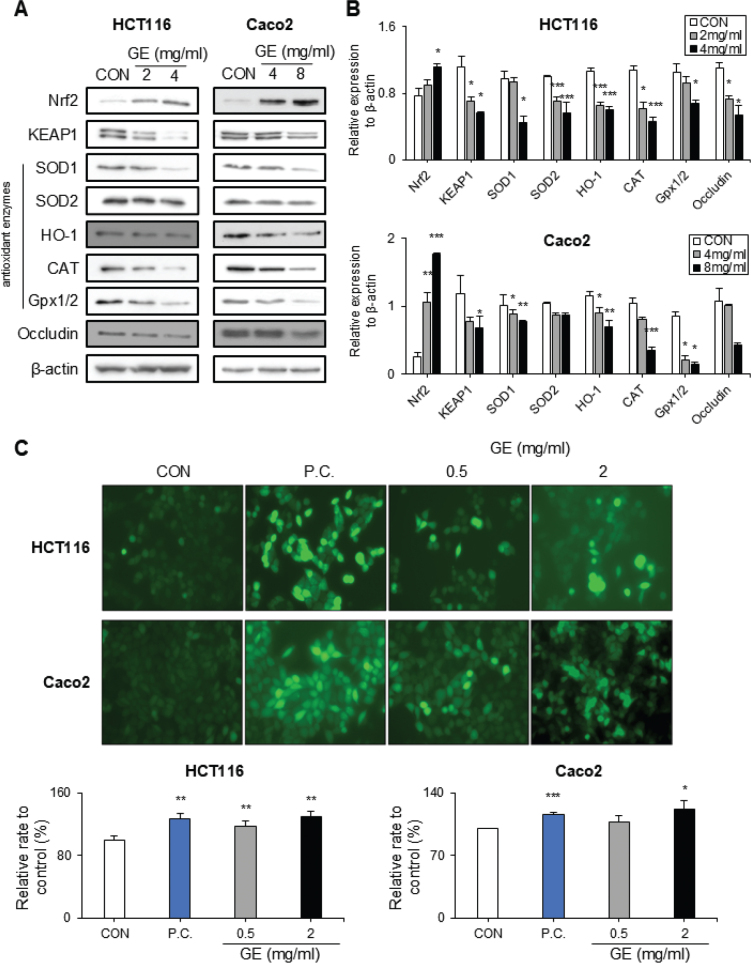
*Gochujang* extract (GE) destroyed the balance of reactive oxygen species (ROS) level and antioxidant proteins in colorectal cancer (CRC) cells. (A–B) HCT116 cells were treated with GE (0, 2, and 4 mg/mL), and Caco2 cells were treated with GE (0, 4, and 8 mg/mL) for 48 h. (A) Western blot band images of proteins associated with ROS metabolism. (B) Relative protein expression of ROS metabolism. (C) The levels of ROS were measured in HCT116 and Caco2 cells after treatment with GE (0, 0.5, and 2 mg/mL) at 24 h. TBHP was used as a ‘P.C.’ Values are mean ± SEM (standard error of the mean) (*n* = 3). * (*P* < 0.05), ** (*P* < 0.01), *** (*P* < 0.001). *t*-Test was performed to evaluate the statistical significance of differences. β-Actin was used as a loading control.

To scrutinize the effects of GE on the ROS metabolism of CRC cells, the level of key antioxidant enzymes was evaluated. Under stressful conditions (e.g. increased ROS level), nuclear factor erythroid 2-related factor 2 (Nrf2) is activated, leading to a decrease of Kelch-like ECH-associated protein 1 (KEAP1), and this process allows Nrf2 to translocate into the nucleus, upregulating the expressions of antioxidant enzymes (44). GE significantly upregulated the expression of Nrf2, whereas the levels of occludin and KEAP1 were decreased in both cell lines, with and/or without statistical significance (Fig. 4A and 4B). Moreover, GE strongly reduced the expressions of major antioxidant enzymes, including SOD1, SOD2, HO-1, catalase, and Gpx1/2 in both CRC cells dose-dependently (Fig. 4A and 4B).

Next, as GE markedly reduces antioxidant system-related protein expressions, the effects of GE on ROS production were measured. As expected, GE markedly increased ROS levels in both CRC cells (Fig. 4C).

Therefore, these observations suggest that GE disrupts the oxidative stress defensive system and increases ROS generation, leading to the imbalance of ROS metabolism in CRC cells.

## Discussion

The worldwide incidence of CRC remains consistently high, especially a recent trend, a high prevalence at younger ages (3). Among the diverse causal factors of CRC, diet can accelerate CRC but prevent/ameliorate CRC (12). Although previous studies addressed the multiple health-beneficial effects of *Gochujang*, including anticancer effects, the detailed cellular and molecular mechanisms of its anticancer effects were not fully investigated. The current study found that GE strongly decreases cell viability, migration, and colony formation. Moreover, GE markedly induces cell cycle arrest and inhibits cell proliferation and cell cycle progression. Additionally, GE also increases apoptosis- and autophagy-related proteins in CRC cells. Lastly, GE alters ROS metabolism in CRC cells by reducing antioxidant enzyme expressions.

Targeting cancer cell viability, migration, and colony formation has been applied in many previous studies when the anticancer effects of certain foods and bioactive compounds were evaluated (17, 45, 46). The present study found that GE significantly reduces CRC cell growth, migration, and colony formation, confirming its anticancer effects against CRC. Interestingly, the effects of GE in CRC cells differed based on the cell lines; for instance, HCT116 cells were the most sensitive to GE among three different CRC cells. These cell line-dependent results are likely to be attributed to the unique characteristics inherent to each cell line (47, 48), indicating the limitations of *in vitro* study. Therefore, to address these limitations of *in vitro* study, future studies need to be performed with *in vivo* models, to confirm the anticancer effects of *Gochujang* against CRC.

Particularly, due to the distinctive ROS metabolism of cancer cells, ROS metabolism of cancer cells can be a targetable therapeutic approach (20). Under the increased oxidative stress environments, Nrf2 is activated to increase the expression of various antioxidant enzymes, including HO-1 and catalase (44). In addition, the decrease in occluding expression also indicates oxidative stress induction in epithelial tissues (49). The present study found that GE significantly increased Nrf2 expression and decreased occludin levels, suggesting GE induces oxidative stress in CRC cells. However, this study showed a reduction in the expression of antioxidant enzymes. This suggests that *Gochujang* might influence on the antioxidant response element (ARE), an intermediary between Nrf2 and antioxidant enzymes (50). Therefore, future studies are required to investigate the effects of *Gochujang* on detailed mechanisms, such as Nrf2/ARE or ARE/HO-1 in CRC cells. Furthermore, this study observed that GE strongly decreased the levels of antioxidant enzymes in CRC cells and increased ROS production, implying GE elevates oxidative stress in CRC cells. These observations indicate that GE induces imbalance of ROS metabolism and oxidative stress, leading to cell death in CRC cells. A previous study found that Manuka honey decreases Nrf2 expression when it causes an interruption of ROS metabolism in CRC cancer cells (23, 51), which was the opposite result of the present study. Thus, further mechanistic studies are required for a better understanding of the effects of GE on ROS metabolism in various cancer cells, including CRC cells.

**Fig. 5:**
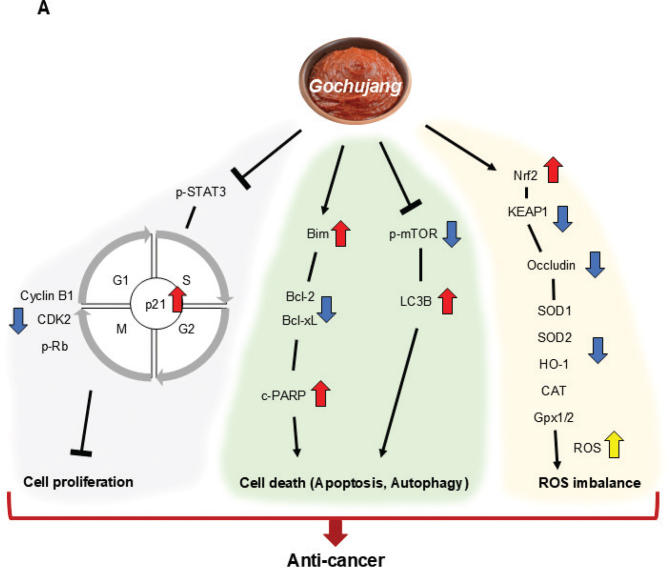
Summary graphic image of the anticancer effects of *Gochujang* extract (GE) in colorectal cancer (CRC) cells.

In conclusion, the current study reports that *Gochujang* has anticancer effects in CRC cells by intergrading various cellular and molecular pathways, including cell cycle arrest, apoptosis, autophagy, and ROS metabolism (Fig. 5). Moreover, this study investigated the effects of GE from whole food, not specific bioactive compounds, providing an understanding of the anticancer mechanisms of whole food. However, in the future, the exact compounds in *Gochujang* providing its anticancer effects should also be investigated and established. Moreover, in the future, it is meaningful to explore the effects of *Gochujang* on other cancer cell metabolism, such as energy metabolism.

## Supplementary Material



## Data Availability

All data presented in the study are made publicly available at or before the time of acceptance.
