# Supplementary figures and images for: Gochujang suppresses cell survival and changes reactive oxygen species metabolism in colorectal cancer cells
Source: Food Nutr Res. 2024 Oct 21;68:10.29219/fnr.v68.10844. doi: 10.29219/fnr.v68.10844 (PMC11549730; doi:10.29219/fnr.v68.10844)

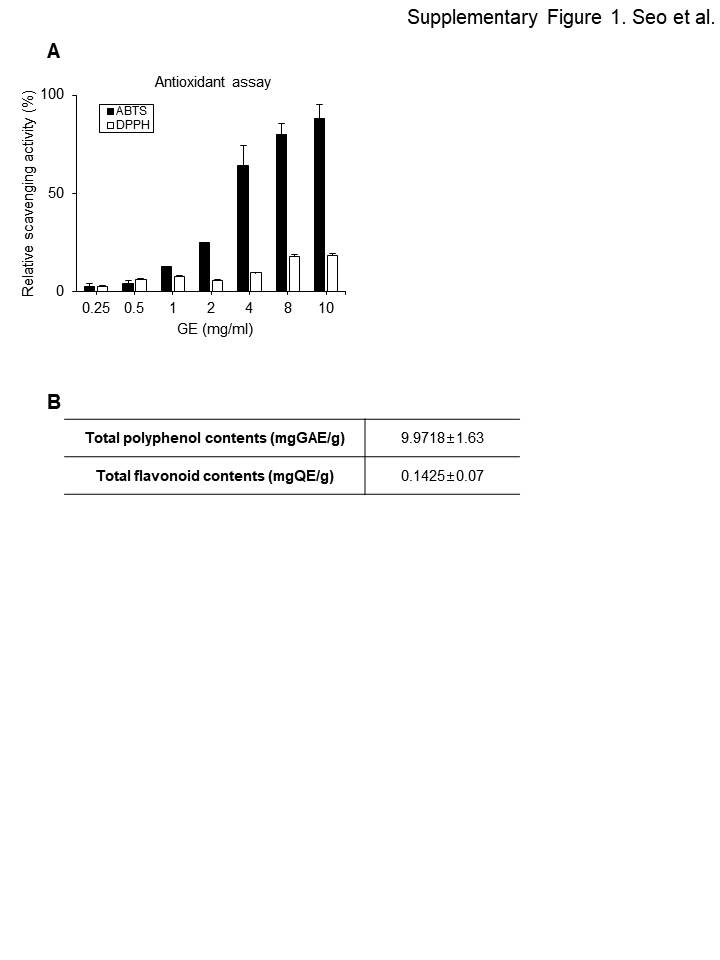


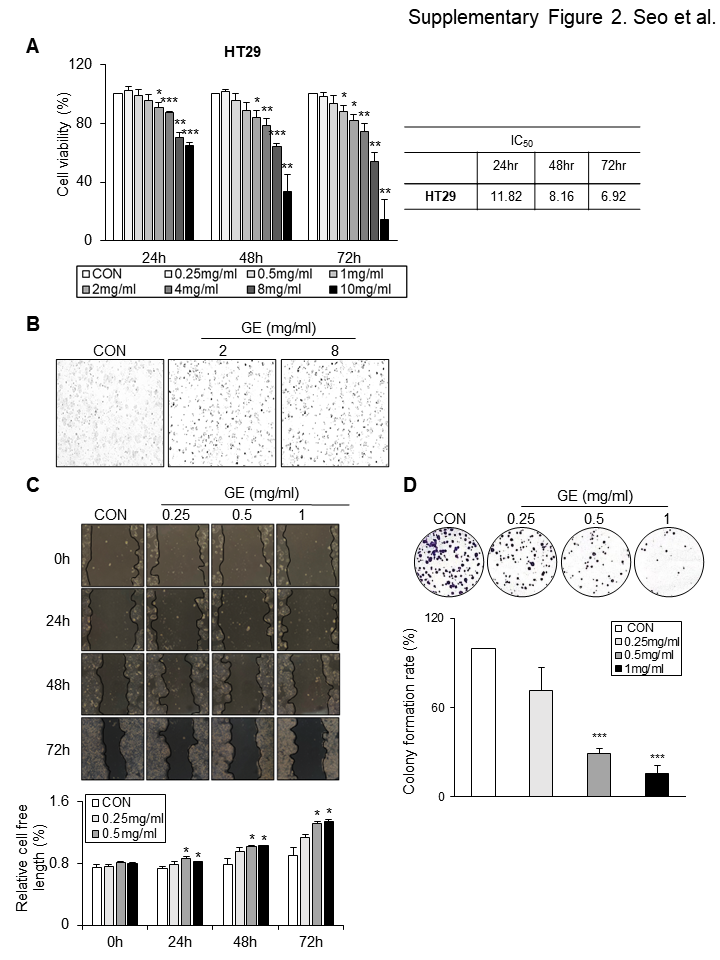


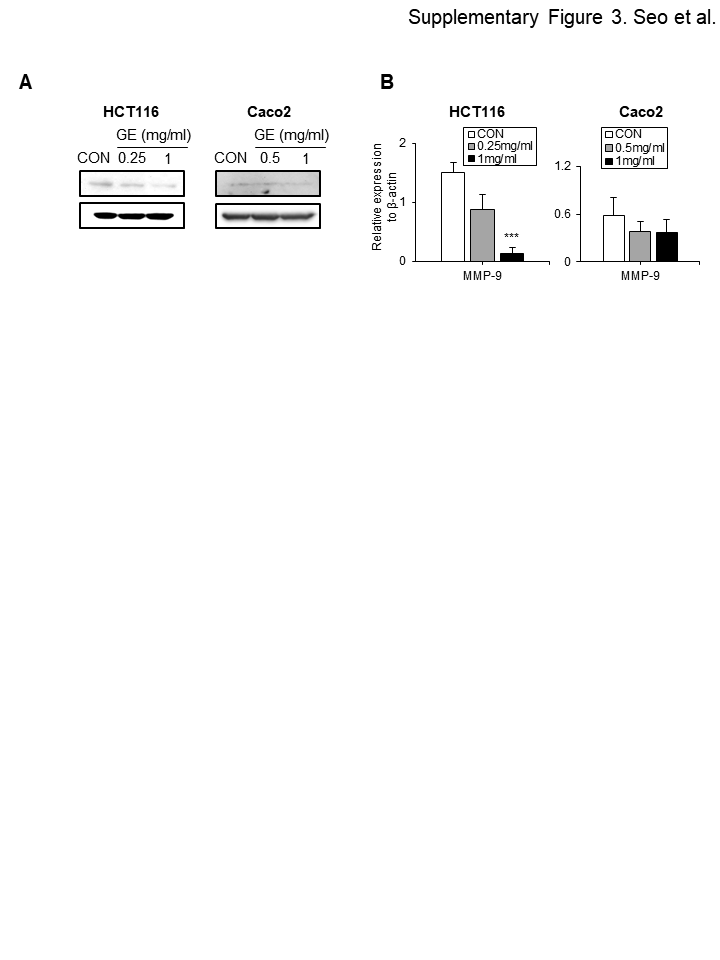

Supplement: Supplementary file 1 [file FNR-68-10844-s1.docx]
